# Editorial: Engaging health systems to address intimate partner violence and advance women’s sexual and reproductive health and human rights

**DOI:** 10.3389/frph.2026.1829453

**Published:** 2026-04-28

**Authors:** Courtenay Sprague, Shelley Brown

**Affiliations:** 1 Department of Conflict Resolution, Human Security, and Global Governance, John W. McCormack Graduate School of Policy and Global Studies, Boston, MA, United States; 2 Department of Nursing, College of Nursing and Health Sciences, University of Massachusetts Boston, Boston, MA, United States United States; 3 Sargent College of Health and Rehabilitation Sciences, Boston University, Boston, MA, United States

**Keywords:** domestic violence, global, health systems, interventions, intimate partner violence (IPV)

Every day, health systems across the world respond to the needs of women exposed to intimate partner violence (IPV). IPV refers to any behavior within an intimate relationship that causes physical, psychological or sexual harm. Affecting nearly one in three women, globally, IPV's burden represents a global public health and human rights problem of great magnitude (1). Significantly, health professionals and systems can and do offer timely interventions that prevent violence and further harm to IPV-exposed women. Serving as interlocutors for women, health providers navigate dynamic social institutions to advance women's fundamental mental and physical health (2). Notably, providers enact agency in ways that, in turn, enable women's agency over their health and risks (3, 4). Moreover, health providers are duty-bearers positioned to protect women's human rights and to advance their human security (5). Ultimately, research indicates that when they function optimally, health systems can become transformative social systems of care and support for women (6). Yet, globally, evidence indicates these opportunities to prevent IPV, and to reduce the risk of further harm, including femicide (7), are insufficiently grasped, even as distinct research gaps remain (8–10). This special research topic is positioned within that opening.

As co-editors, our aim was to leverage global research to inform national and global policies and clinical practice where possible: from addressing health inequities for key affected populations of women (SDG 3), ensuring SRH (SDG 3.7), promoting gender equality (SDG 5), and importantly, eliminating all forms of violence against all women and girls in both public and private spheres (SDG 5.2.1) (11). Here, we highlight principal knowledge contributions from the 8 published articles in this collection, representing the research of over 50 authors. Their work examines the dynamic interventions, policies, outcomes and considerations unfolding in these global contexts. Next, we look across and within these papers to identify cross-cutting themes and novel contributions across the collection. We close with mention of several possibilities for further research.

Much of the evidence of “what works to prevent violence” has been gathered in community settings via short-term randomised controlled trials, which are critical, yet difficult to scale. Hence, multi-sectoral approaches to IPV, given the presence of government leadership and policy, enable scale, enhance health equity and financing (also solving the sustainability problem) [see (12, 13)]. To that end, two publications had national governments—Kenya and Rwanda—as essential partners in their study interventions (Doyle et al.; Menzel et al.).

In Kenya, Menzel et al. authored the first known published study of a government adopting provider training and guidelines that integrate reproductive coercion into family planning services via the “Addressing Reproductive Coercion in Health Settings” (ARCHES) intervention. Partners sought to address policy goals that included reducing gender-based violence and unintended pregnancy while advancing women's reproductive autonomy. The ARCHES intervention integrated universal education, screening and support on reproductive coercion and IPV during routine family planning counselling, with a view to adaptation and scale, nation-wide. This study exemplifies how evidence-based interventions and implementation strategies can be achieved through effective partnerships, though such interventions necessitate careful adaptation to align with social context, and likely, continuing political will.

In Rwanda, Doyle et al. assessed the impact of a training on more than 550 community health workers (CHWs) and their capacity to deliver Bandebereho (role model) —a parenting programme—during scale up in Burera District. They relied on critical reflection of gender norms and understanding of power, harnessing a theory of change, where creating safe spaces for men and their partners to reflect on the costs of strict gender norms might enable participants to learn and practice more equitable attitudes and relationship skills, culminating in benefits across a range of health and relationship behaviors. Findings stressed the importance of investing in high-quality facilitator training to ensure sufficient time for facilitators' own transformation, to maintain quality and fidelity at scale, further emphasizing a 'slow and steady' approach for adaptation, testing and refining IPV programmes at scale—to support government ownership. By post-survey and follow up, CHWs reported improved relationships with partners, greater partner support, and also at follow up, a marked degree of comfort and confidence to implement the Bandebereho intervention. This work yields insights concerning the subsequent, multiple benefits for the personal relationships of CHWs and their work that is relevant across settings.

Climate changes inordinately affect women and girls, functioning as a “trigger” for intimate partner violence, through stresses on the livelihoods of communities. At the same time, climate stresses and their effects constrain opportunities for SRHR among girls and women in Rukiga, Uganda, write Mayhew et al. While these problems are connected, cross-sectoral approaches to health services, secure livelihoods and environmental protection remain largely segregated. Employing a qualitative paradigm, the authors investigated whether integrated programmes could improve IPV, SRH and gender equity in Uganda, comparing the delivery of a suite of programmes pre- and post-intervention (Apr 2021-Dec 2023). Over 44 focus groups, the authors richly document participants' lived experiences of food insecurity, poverty and IPV. The authors found that such programmes can advance women's reproductive health, family planning and choice, while addressing root causes of IPV: abuse and coercion by partners. Nonetheless, the authors emphasize in climate-affected regions, particularly, success will rest upon greater integration across social services (in programming and delivery) with careful attention to gender-justice and power and resources tailored to social-structural context.

Young people, globally, and in sub-Saharan Africa (SSA), face impediments to realizing their sexual and reproductive health and rights, including adverse social norms that influence behavior. Leite et al. conducted a scoping review of social norms interventions in SSA that improve SRHR outcomes (40 interventions from 12 countries) among 10- to 24-year-olds—including reducing IPV and GBV. Marked absences they uncovered included clear diffusion strategies to disseminate new norms and behaviors, and a lack of implementation research to inform scaling and sustainability of social norms interventions. The authors examined interventions across eight programmatic strategies and six SRHR outcomes to illuminate key insights, synthesizing content, definitions and designs, modes of delivery and evidence for impact in ways that underscore that overturning adverse social norms is indeed possible. These interventions illuminate how.

IPV-exposed women are at high risk of injuries, making emergency departments and trauma centers obvious contact points for women. Yet adoption of IPV screening in hospital trauma centers has not matched this reality, even in Canada. Accordingly, Montesanti et al. investigated how providers in two trauma centers of Edmonton, Alberta Province, perceive and enact IPV screening, with attention to cognitive processes, barriers, and facilitators to implementation. The authors utilized cognitive task analysis (CTA), the Consolidated Framework for Implementation Research (CFIR) and Proctor's taxonomy of implementation outcomes in their study to unmask the knowledge, processes of decision-making and cognitive demands of providers navigating these high-stress environments. They organized their findings into six cognitive domains. By pinpointing cognitive barriers, the authors' work contributes novel findings on the improvement of such health services through implementation strategies that seek to integrate IPV screening into trauma care via evidence-informed practices, with greater feasibility and sustainability.

Also from Canada, Villacis Alvarez et al. shed important light on organizational and systems-level barriers and facilitators shaping health professionals' readiness to address domestic and sexualized violence in Nova Scotia, a province that has declared IPV an epidemic and recorded femicide rates above the national average. Drawing on a mixed-methods survey of 1,649 health professionals, the authors qualitatively analyzed open-ended responses from 828 participants using reflexive thematic analysis within a feminist poststructuralist framework, attending to how institutional discourses construct meaning and shape providers' sense of role and responsibility. Two themes emerged: first, deeply inconsistent approaches to addressing violence, wherein health professionals drew on contrasting discourses to define whether violence fell within their scope of practice. Some embraced holistic, trauma-informed frameworks and others relied on strict biomedical constructions of clinical care. Second, participants described the frustrating limits of a downstream health system ill-equipped to address the structural conditions, including housing insecurity, food precarity, and fragmented social supports. These determinants underpin and perpetuate violence, as well as inequities further exacerbated by the COVID-19 pandemic. The authors call for clearer governmental and organizational policies on scopes of practice, sustained training, and meaningful intersectoral coordination to build a health system response that moves upstream to address the root causes of violence.

Notably, responses in health settings can emphasize immediacy, while overlooking the chronic toll of IPV and its underlying causes—not least adverse gender norms and social-structural determinants of health—as Mantler and Wathen stress in their perspective piece (2026). They propose and explicate their original conceptual and practical approach: trauma- and violence-informed care (TVIC). TVIC has the advantage of integrating, and going beyond existing trauma theory, in ways that attend to the pernicious social inequities rooted in the fabric of societies and their consequences. TVIC effectively integrates safety, choice and trustworthiness to enable service offerings to survivors to be attuned to these complexities in ways that are equity oriented, while seeking to dismantle barriers that women confront in health and other settings.

Perinatal populations are at greater risk of IPV exposure. Neff et al. investigated the relationship between IPV and domestic violence (DV) among Black and White birthing persons during pregnancy, given restrictive abortion policy in selected US states. They analyzed (2020) data gathered from 36 of 50 states using the US CDC Pregnancy Risk Assessment Monitoring system (encompassing nearly two million deliveries by women), paired with the Guttmacher Abortion Policy Hostility Index. The authors found those persons residing in states with more restrictive abortion access were more likely to report IPV/DV during pregnancy. By implication, restrictive policies can magnify existing health inequities, particularly among already disadvantaged individuals, especially Black perinatal populations in the United States. Conversely, laws and policies that recognize and support women's SRHR can be protective against IPV and support their holistic health.

Collectively, these eight papers employ a diverse methodological toolkit to advance understandings of IPV and SRHR and the structural conditions shaping both. They range from a large-scale scoping review of 40 experimental and quasi-experimental interventions across 12 sub-Saharan African countries (Leite et al.) to pre/post longitudinal cohort designs assessing community health worker training (Doyle et al.) to weighted logistic regression applied to population-level surveillance data across 36 US states (Neff et al.). Qualitative and mixed-methods approaches are also well represented, including focus group discussions tracking lived experience over time in Uganda (Mayhew et al.), reflexive thematic analysis within a feminist poststructuralist framework in Nova Scotia (Villacis Alvarez et al. 2026), and systematic adaptation-tracking using FRAME + IS to document intervention modifications within a public health system (Menzel et al.). Mantler and Wathen's perspective piece contributes a robust conceptual framework, Trauma- and Violence-Informed Care (TVIC), that explicitly names structural and intersecting oppressions as drivers of harm, complementing the empirical work across the other papers. Together, these contributions span intervention design, scale-up fidelity, health system integration, and structural policy analysis, offering both practical tools and theoretical grounding for researchers and practitioners working at the intersection of gender, health, and violence prevention.

Several important gaps and tensions emerge across this body of work. Most striking is the persistent silo problem: programmes addressing IPV, SRHR, climate stress, livelihoods and reproductive coercion tend to be designed and evaluated in isolation, even when the evidence, most clearly represented in the work by Mayhew et al. and Menzel et al., demonstrates that integrated, cross-sector delivery produces more meaningful outcomes.

A second recurring gap is the absence of diffusion and sustainability strategies. Leite et al. explicitly flag that most social norms interventions focus on awareness without clear pathways for norm spread, and Doyle et al. raise similar concerns about maintaining fidelity at scale (2025). Menzel et al. and Villacis Alvarez et al. (2026) both highlight the tension between evidence-based intervention design and implementation realities: adaptation is inevitable and sometimes politically constrained, yet systematic documentation of those trade-offs remains rare. Neff et al. stand out as the lone study examining policy-level structural drivers—specifically abortion restrictions—finding that restrictive state policy is associated with elevated IPV odds, particularly among Black birthing individuals. Thereby introducing a legislative and racial equity dimension unique to the US context.

Overall, palpable gaps in research, policy and practice remain. Limitations in the availability and quality of data and in our understanding of health system responses, as well as women's experiences in health settings, persist. Greater research attention to geographic locations that remain underrepresented in the literature is critical, including Latin America and the Caribbean, Central and South Asia and the Pacific Island nations, while recognizing that these regions, and that of regional organizations like the Pan-American Health Organization (PAHO), have moved the field forward (14). Neglected settings also encompass humanitarian, conflict, and post-conflict contexts where IPV prevalence is elevated but health system capacity is typically severely constrained.

Governance and policy frameworks that could gain traction on addressing IPV in health systems, including at the local and national levels are needed. At the nexus of law, justice and health systems, greater research could be undertaken concerning how a range of health laws and policies (e.g., firearms) can be protective or restrictive, whether promoting (or diminishing) women's SRHR; and the ways health systems manage and navigate these, such as through medical-legal partnerships that advance women's human rights [see (15)].

The essential need for greater attention to key populations is also manifest. This encompasses women excluded and marginalized in their societies, particularly those experiencing multiple, intersecting forms of discrimination, such as women with disabilities and particularly LGBTQIA + at highest risk of IPV. The need to re-formulate and document conceptualizations of gender is evident, including gender identity, to better understand the experience of non-binary populations and influences on health and wellbeing for IPV-exposed populations. Theoretically, the field would benefit from more explicit engagement with intersectionality as an analytic framework rather than a descriptive lens, moving beyond acknowledgment of overlapping vulnerabilities toward structural explanations of how race, class, gender, and coloniality jointly produce differential exposure to IPV and unequal access to care.

Notably, mental health is largely absent as an explicit outcome of focus across these papers, despite robust evidence linking IPV exposure to depression, anxiety, PTSD, and other psychological harms. Wathen and Mantler’s perspective pieces comes closest, foregrounding trauma and the risk of re-traumatization within care systems through its TVIC framework. Villacis Alvarez et al. also, helpfully, allude to the cumulative psychological burden on both survivors and health professionals navigating structural barriers. Given that psychological wellbeing is both a consequence of IPV and a determinant of whether survivors can access and benefit services—including those provided in some of these studies—this domain demands greater research attention.

The psychological and professional toll on health professionals is a critical area for future work. Secondary traumatic stress and burnout among those responding to IPV are well-documented phenomena. Additional research could address provider mental health and burnout as an implementation consideration due to its direct bearing on the quality, consistency and sustainability of care that survivors receive.

Emerging digital and technology-based approaches, including mobile health applications, digital screening tools, and media-based norm change programmes, appear across several papers in this collection. However, they remain undertheorized as a distinct intervention modality and represent a clear domain for further research. To date, the evidence base for digital health tools in IPV prevention and response remains thin, and questions of equitable access, data privacy, and safety for survivors using such tools are largely unresolved. Given the rapid trajectory of digital health innovation, this represents both a timely and urgent opportunity.

Methodologically and ethically, longitudinal and mixed-methods designs that can capture norm change over time remain underdeveloped, as does community-based participatory research that positions affected populations as co-investigators rather than subjects. The voices and agency of adolescents, survivors and community members are more often objects of study than co-producers of knowledge, pointing to a broader methodological opening for more participatory and community-led research designs.

Ethical approaches to engage with participants in IPV-related research require sustained interrogation, with concerted focus on how participants are involved in such studies as partners. Given the preponderance of Global North journals and accompanying health institutions, funding and evidence, decolonizing research and methods and continued scrutiny on such power imbalances remains a principal task of IPV and related global health research. Taken together, these gaps underscore that while this collection advances the field meaningfully, the work of building health systems that are truly responsive to IPV-exposed women remains substantial and pressing.
